# Evaluation of the Effect of Laser Acupuncture and Cupping with Ryodoraku and Visual Analog Scale on Low Back Pain

**DOI:** 10.1155/2012/521612

**Published:** 2012-10-11

**Authors:** Mu-Lien Lin, Hung-Chien Wu, Ya-Hui Hsieh, Chuan-Tsung Su, Yong-Sheng Shih, Chii-Wann Lin, Jih-Huah Wu

**Affiliations:** ^1^Institute of Biomedical Engineering, National Taiwan University, No. 1, Section 4, Roosevelt Road, Taipei City 10617, Taiwan; ^2^Department of Pain Management, Taipei City Hospital Zhong Xing Branch, Zheng Zhou Road, No.145, Taipei 103, Taiwan; ^3^Yi Sheng Chinese Medicine Clinic, Room 3, 4 Floor, No. 333, Fuxing N. Road, Songshan District, Taipei City 105, Taiwan; ^4^Department of Healthcare Information and Management, Ming Chuan University, No. 5, Deming Road, Gweishan Township, Taoyuan 333, Taiwan; ^5^Department of Biomedical Engineering, Ming Chuan University, No. 5, Deming Road., Gweishan Township, Taoyuan 333, Taiwan

## Abstract

The purpose of this study was to evaluate the effect of laser acupuncture (LA) and soft cupping on low back pain. In this study, the subjects were randomly assigned to two groups: active group (real LA and soft cupping) and placebo group (sham laser and soft cupping). Visual analog scale (VAS) and Ryodoraku were used to evaluate the effect of treatment on low back pain in this trial. Laser, 40 mW, wavelength 808 nm, pulse rate 20 Hz, was used to irradiate Weizhong (BL40) and Ashi acupoints for 10 minutes. And the Ryodoraku values were measured 2 times, that is, before and 15 minutes after treatment. The results show that there were significant difference between the first day baseline and the fifth day treatment in VAS in the two groups. Therefore, LA combined with soft cupping or only soft cupping was effective on low back pain. However, the Ryodoraku values of Bladder Meridian of the placebo group have been decreased apparently, and didn't come back to their original values. It means that “cupping” plays the role of “leak or purge” in traditional Chinese medicine (TCM). On the other hand, the Ryodoraku values of Bladder Meridian of the active group have been turned back to almost their original values; “mend or reinforcing” effect is attributed to the laser radiation.

## 1. Introduction

Most people often disregard the severity and the impact of low back pain (LBP). However, the influences of LBP can be very widespread, especially in the aspect of quality of life. And the impact of LBP will lead to spinal instability finally. It will produce more uncomfortable status and lead to chronic LBP eventually [1].

Acupuncture was the oldest and also an important therapy of TCM. It has been accepted for pain relief, and it was regarded as a complementary therapy in most countries [2]. At present, there was sufficient evidences to prove the clinical value of acupuncture [3, 4] and encourage further studies to elucidate the relationship between physiological changes and clinical outcomes. For instance, needle acupuncture has 80% subjective improvement on osteoarthritis of the knee [5]. Moreover, various studies have shown that needle acupuncture has efficacy for the treatment of long-term disease of neck pain [6]. Ahsin et al. also showed that the electroacupuncture active group was more effective than the placebo group for improvement of stiffness and disability on osteoarthritis of the knee [7]. However, for those people who were afraid of needles, they do not will to endure the tingling of acupuncture. Some researchers tried to replace the needles by using laser; therefore, it was called "laser acupuncture" (LA). LA has the characteristics of being noninvasive, noninfectious, easy to use, and it can avoid the pain and psychological fear of traditional acupuncture. Thus, LA was chosen in this trial.

After laser biostimulation was published by a Hungarian professor, Dr. Mester, in 1969, low level laser therapy (LLLT) has gradually gained popularity from eastern Europe to the whole world. Many scholars also used Nd:YAG laser or semiconductor diode laser as laser source to treat lower back pain and musculoskeletal back pain [8, 9]. In 2002, Molsberger et al. showed that acupuncture (fixed points) plus conventional orthopedic therapy versus sham plus conventional orthopedic therapy was statistically significant (*P* = 0.013) after test [10]. LA was widely used for treatment of acute or chronic pain, such as chronic myofascial pain in the neck [11]. Shen et al. showed that the pain of osteoarthritis was reduced on active laser treatment group [12]. There was another therapy called cupping, and it can remove the wind-cold-dampness, stagnant blood. In addition, the combination of acupuncture and cupping was an appropriate therapy with a shorter treatment course [13]. After conducting the LA and cupping in the painful area, it can facilitate the flow of QI in meridians. According to Arndt-Schulz Biological Law [14], when energy densities were too small, no significant effect can be observed. Higher energy densities resulted in the inhibition of cellular functions. Thus, low energy laser was used in this study.

The Ryodoraku (meridian) theory was developed by Nakatani [15], and the values of Ryodoraku can reflect the conditions of the relative meridians and organs by analyzing and comparing their changes with microelectrical current. The Ryodoraku gives a clear definition of its measuring. The electrical current between two acupoints was larger than 90 *μ*A or smaller than 50 *μ*A, and it represented their the relative meridian is excess syndrome or deficiency syndrome, respectively. In 1998, Ulett et al. noted that acupuncture includes many techniques such as acupressure, shiatsu, laser acupuncture, Ryodoraku, electro-acupuncture, and more [16]. In 2003, Wang et al. used acupuncture with Ryodoraku for hypertension patients, and the result indicated that the Ryodoraku value, blood pressure, and pulse rate were reduced after stimulating at Zusanli acupoints [17]. In the study of Sancier, the subjects practiced QI gong approximately five hours for two days, and the results revealed that the balance of improvement of body energy for the group was observed through the Ryodoraku value [18]. In 2005, Weng et al. used Ryodoraku to evaluate the effect of tennis elbow pain and back pain [19, 20].

 Furthermore, the LA plus soft cupping on the efficacy of back pain has not been published yet. And to measure the Ryodoraku of the meridians of the subjects is a good way to understand the variation of meridians before and after using laser acupuncture and soft cupping [15]. Thus, in this study, low level laser acupuncture and soft cupping were used to stimulate the patient's acupoints, and Ryodoraku and VAS were used to evaluate the improvement of the symptoms of chronic LBP. 

## 2. Materials and Methods

### 2.1. Subjects

A total of 60 patients of either sex with LBP for at least three months were recruited in the study from Taipei Municipal Chung-Hsin Hospital. Ethical approval was granted by Taipei Municipal Chung-Hsin Hospital ethical committee. All the patients were diagnosed by a doctor. The patients with other complications like heart attack, kidney problem, including pregnancy, were excluded from this study. They were randomly assigned to active group (real LA with soft cupping) and placebo group (sham LA with soft cupping). After the diagnosis, each patient included in the study was explained the procedure of study. Written informed consent was taken, and relevant history of each patient was recorded.

### 2.2. Procedure

**Figure 1:**
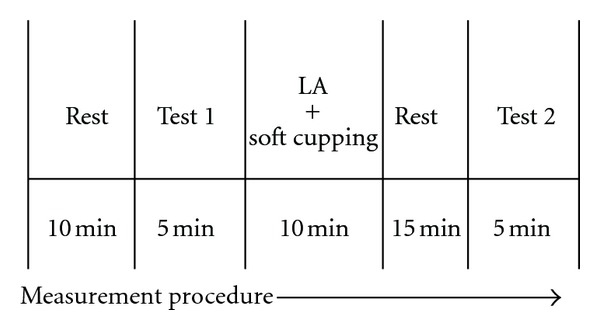
Protocol in this study.

**Figure 2:**
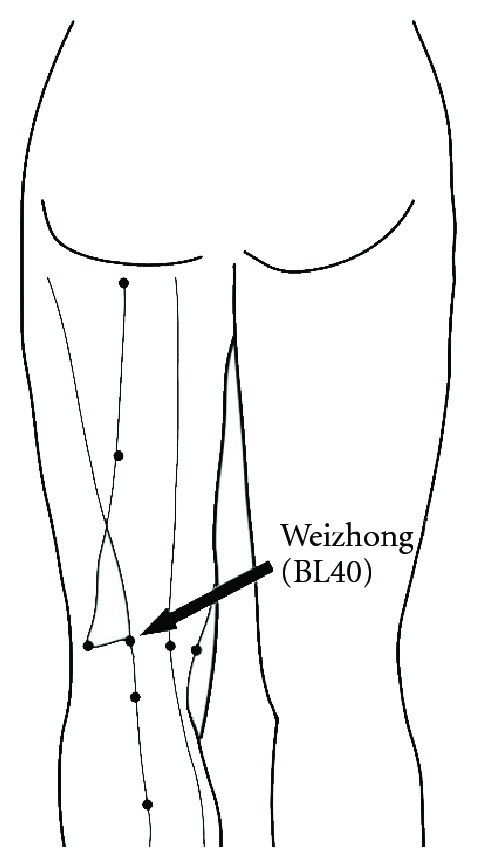
Weizhong (BL40) acupoint.

**Figure 3:**
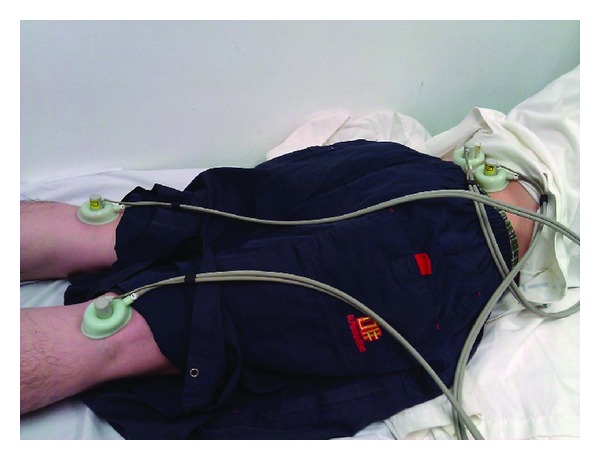
The treatment combining LA with soft cupping in this study.

All patients lied down on the bed in the room air-conditioned (25°C) and kept quiet. The protocol in this study was followed in Figure 1. Every patient received one treatment (five continuous days). First, we recorded the visual analog scale and measured the Ryodoraku values for all patients before the trial. Second, we used 4-channel laser therapy instrument LA400 (manufactured by United Integrated Services Co., Ltd., Taiwan) to treat Weizhong acupoints (BL40) on two feet and Ashi points on dorsal for 10 minutes, see Figures 2 and 3. The sham group has the same procedure as the laser group, however, without laser radiation. The two groups also received soft cupping treatment at the same time. After treatment the patients took a break of about 15 minutes; finally, we recorded the VAS and measured the Ryodoraku values again.

### 2.3. Instrument

The depth of penetration of laser varies with wavelength. Generally, near infrared light has deeper tissue penetration than that of the visible light. The laser therapy instrument LA-400 that operated with a pulsed laser beam (40 mW output power, wavelength 808 nm, spot size 0.8 cm^2^, pulse rate 20 Hz, 50% duty cycle of the pulse, 10 minutes treatment) was used in this study. Thus, the dosage in this study is approximately 15 J/cm^2^.

### 2.4. Measurement

A Ryodoraku measurement device, Health Director, Model HD747, manufactured by Nay Yuan technology Co., Ltd. in Taiwan, was used in this study. According to the instruction, when the current was less than 50 *μ*A, it represented that the relative meridian was “deficiency” syndrome, when the current was greater than 90 *μ*A, it represented that the relative meridian was “excess” syndrome. The average and standard error of such values were determined and expressed as “Mean ± STD” for statistical analysis.

### 2.5. Statistical Analysis

The scores of VAS in each group before and after the treatment were compared with paired-sample *t*-test. The 12 meridians, lung, pericardium, heart, small intestine, triple energizer, large intestine, spleen, liver, kidney, bladder, gallbladder and stomach, of groups were analyzed by using the paired-sample *t*-test. The difference between the values of patients in the two groups measured before and after treatment was analyzed. All the statistical tests were two-tailed. A statistical significance was recognized as *P* value <0.05.

## 3. Results

**Figure 4:**
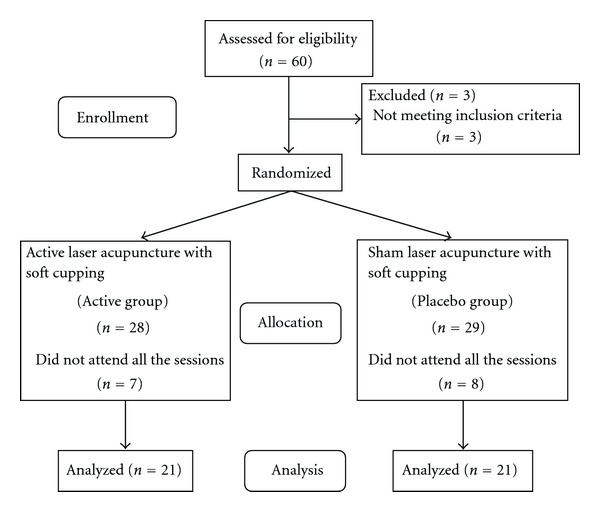
Consort flow diagram.

In this study, 60 patients who have chronic LBP were recruited, in total. 28 patients were recruited for active group of which 21 completed the study protocol. In the control placebo (or sham) group, 29 patients were recruited in total, but failure to complete was 8, see Figure 4 for demographic details. There was no significant difference of age, weight, height, and BMI between the two groups as shown in Table 1. After the collection of data, we compared and analyzed the value of active group and placebo group. Baseline measurements of 12 meridians showed that there were no significant differences between these two groups as listed in Table 2.

**Figure 5:**
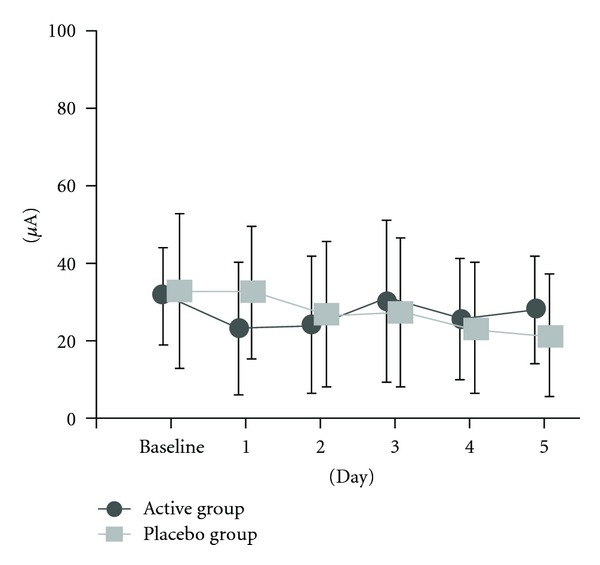
The change of Ryodoraku value of Bladder Meridian after treatment in two groups.

The result reveals that the Ryodoraku values of 12 meridians were reduced by cupping in both groups. In active group, most of the Ryodoraku values of 12 meridians were reduced in the first day due to the combination of LA and cupping. On the fifth day, most of the Ryodoraku values of 12 meridians were raised back to almost original values due to the irradiation of laser light. However, in placebo group, most of the Ryodoraku values of 12 meridians were also reduced on the first day, but not obviously. On the fifth day, most of the Ryodoraku values of 12 meridians were not raised back to original values without the irradiation of laser light. In this study, Weizhong acupoint (BL40) which belongs to bladder Meridian was chosen. It is worthy to mention the variations of Ryodoraku value of Bladder Meridian after treatment in two groups. In active group, the Ryodoraku value of Bladder was raised back on the fifth day, but it has not been raised back in placebo group. It means that “cupping” plays “leak or purge” role in TCM, as shown in Figure 5. And laser acupuncture plays “mend or reinforcing” role in TCM.

**Figure 6:**
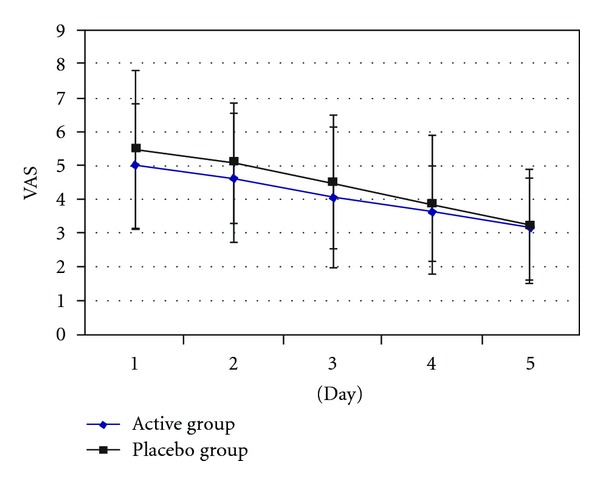
Statistics and analysis VAS of patients after the treatment in two groups.

 The VAS scales of all patients are decreased in these two groups after 5 times of treatment, but it had no significant difference between these two groups in each treatment, see Figure 6 and Table 3. However, it had been statistically significant (*P* < 0.01) for VAS compared with the first day and the fifth day in active group and placebo group, see Tables 4 and 5. The results show that there were significant differences between the first day baseline and the fifth day treatment in VAS in the two groups.

## 4. Discussion

LBP was the most common disease in the world [21]. 70%–85% people suffer LBP at some time in life, and the prevalence per year was 15%–45% [22]. There are many etiologies of LBP, for example, acute or chronic strain, sprain contusion, and degenerative disease of lumbar spine. In our study, most of the patients (42/60) were caused by degenerative disease of lumbar spine and finally disc pain, facet pain, or radicular pain happened; others (18/60) were myofascial pain caused by sprain or strain injury. From TCM point of view, Xiong et al. used factor analysis to explore patterns of symptoms and signs from patients with chronic low-back pain based on the TCM theory. They found that four factors were extracted from LBP patients, including (1) Qi and/or blood stagnation, (2) cold/damp, (3) a part of “kidney deficiency,” and (4) warm/heat [23]. The research of Sherman et al. also showed that Qi and blood stagnation or Qi stagnation was found for 85% of LBP patients and kidney deficiency was found for 33–51% of LBP patients [24].

LA was a kind of phototherapy at acupoint similar to needle acupuncture with different kind of perturbation energy. Low level laser has been used for acupuncture treatment to replace traditional acupuncture, and it had been reported that LA was effective in many diseases, such as osteoarthritis of the knees, a kind of degenerative disease [12]. The near infrared laser range of about 600–1400 nm was the most suitable wavelength for LA, because it can infiltrate the skin 2–5 mm, and dosage can be cumulated if irradiating at the same location. So far, LA combined with soft cupping treatment on LBP has not been reported yet, but previous studies have shown that LA can stimulate acupuncture points [12]. In this study, 60 patients who have chronic LBP were recruited from outpatient visits, and 42 patients completed the trial. We recorded the VAS and measured the Ryodoraku values for all patients before and after the treatment. There was no significant difference between these two groups of Bladder Meridian before LA and soft cupping. But the Ryodoraku value in the active group was decreased apparently; however, on the fifth day, it almost returned to original value after laser irradiation, which might be due to the dosage that was cumulated by LA. Karu et al. noted the continuing effects and delayed effects of laser irradiation [25]. We found that the Ryodoraku value of Bladder Meridian had significantly changed in active group after the treatment, and it had statistically significance on first two days. However, there was no statistically significance of Bladder Meridian on the baseline and the fifth day after treatment due to the effect of laser acupuncture, as shown in Figure 5. In addition, the scores of VAS decreased after the trial in both groups. Besides, the scores of VAS had statistically significance before and after LA and soft cupping on the first day baseline and the fifth day in two groups in this trial. 

According to TCM acupuncture theory, some kind of energy or information worked in our body was called qi. The source points of Ryodoraku were most associated with the internal organs in the body and the energetic level of the meridians. Thus, these points are effective for measuring meridian energy, or QI. In addition, there were some subjects who received acupuncture treatment experienced sensations of soreness for needle acupuncture and obtaining of QI [26]. We found that the QI-blood can be reactivated after irradiated with the laser acupuncture at Weizhong acupoint of Bladder Meridian of Foot Taiyang. According to TCM, cupping on the human body will cause warm and stimulating effect on local skin and improved local blood circulation. It was well known that the Ryodoraku value of Bladder Meridian would be increased based on the complemental functions of TCM. Thus, we thought that the improvement of LA at Weizhong acupoint was similar to the effect caused by traditional acupuncture. From these studies, we do believe that continuous treatment with LA for a consecutive time can improve LBP and influence the relative meridians. And the results indicated that LA combined with soft cupping at the Weizhong acupoint and Ashi acupoint can relieve the symptom of LBP. Therefore, treatment with LA and soft cupping at the Weizhong and Ashi acupoints was effective on LBP.

## 5. Conclusion

After five days, the Ryodoraku values of some meridians decreased significantly in placebo group. On the other hand, most of the Ryodoraku values of twelve meridians changed back to almost original values in active group. The cupping seems can “leak or purge” the relative meridians, its effect on relieving low back pain is positive, but the relative meridians seem to be changed to deficiency syndrome. However, the laser acupuncture can raise the Ryodoraku values of the relative meridians. Hence, the findings in this study found that LA and soft cupping can be a suitable treatment choice for patients with LBP.

## Figures and Tables

**Table 1:** Demographic data for the study groups.

	Age	Weight (Kg)	Height (cm)	BMI (kg/m^2^)
Active (*n* = 21)	63.35 ± 11.23	69.04 ± 12.17	160.00 ± 8.74	26.98 ± 4.33
Control (*n* = 21)	64.65 ± 13.57	64.41 ± 15.50	159.68 ± 9.26	25.07 ± 4.24

**Table 2:** Comparisons between the Ryodoraku values of 12 meridians at baseline and that after therapy from the first day to the fifth day in two groups.

12 meridians *n* = 21	Active	Placebo	Active group					Placebo group				
	Baseline		Combine LA with soft cupping					Soft cupping				
	First day	First day	First day	Second day	Third day	Fourth day	Fifth day	First day	Second day	Third day	Fourth day	Fifth day
	Before		After					After				
Lung	47.55 ± 19.71	46.54 ± 18.71	35.25 ± 20.73^∗^	37.13 ± 21.69	45.80 ± 26.33	35.74 ± 17.30	52.81 ± 24.37	40.24 ± 23.04	32.40 ± 19.65^∗^	34.23 ± 19.81^∗^	31.39 ± 18.71^∗∗^	38.08 ± 2.86
Pericardium	43.47 ± 16.19	40.12 ± 17.22	34.42 ± 19.83	33.59 ± 20.38	41.24 ± 25.18	33.41 ± 19.55	42.48 ± 20.02	36.12 ± 19.65	29.31 ± 17.01^∗^	30.34 ± 17.29^∗^	29.58 ± 18.21^∗∗^	33.50 ± 22.23
Heart	37.84 ± 13.51	35.96 ± 15.81	28.16 ± 16.19^∗^	27.35 ± 16.14^∗^	34.22 ± 16.54	28.30 ± 13.55^∗^	35.44 ± 15.58	31.33 ± 16.93	24.92 ± 15.76^∗^	25.76 ± 16.28^∗^	24.74 ± 15.55^∗∗^	29.73 ± 21.37
Small intestine	45.84 ± 16.61	41.74 ± 18.14	34.85 ± 20.43^∗^	34.47 ± 21.38^∗^	40.13 ± 22.14	35.46 ± 17.94	45.36 ± 21.79	37.11 ± 19.86	28.45 ± 17.30^∗^	30.14 ± 17.79^∗^	29.48 ± 19.86^∗∗^	34.04 ± 21.93
Triple energizer	47.54 ± 22.36	45.08 ± 19.83	39.89 ± 23.45	40.08 ± 22.95	47.04 ± 26.78	39.27 ± 18.25	52.90 ± 27.02	38.18 ± 22.25	31.31 ± 18.96^∗^	34.72 ± 18.94^∗^	29.56 ± 18.36^∗^	37.12 ± 20.08
Large intestine	46.33 ± 20.28	46.48 ± 19.65	29.23 ± 21.28	38.34 ± 20.22	47.17 ± 26.00	36.17 ± 14.98	50.29 ± 21.21	37.82 ± 22.04	30.60 ± 18.93 ^∗∗^	34.71 ± 17.20^∗^	31.77 ± 18.67^∗∗^	35.24 ± 18.39^∗^
Spleen	36.86 ± 13.3	38.42 ± 23.62	28.02 ± 19.37^∗∗^	30.76 ± 20.70	35.94 ± 24.89	29.53 ± 15.82	34.73 ± 19.97	29.17 ± 13.47	24.67 ± 15.31	22.03 ± 12.40^∗∗^	24.12 ± 15.40^∗^	24.65 ± 13.55^∗^
Liver	34.96 ± 15.12	33.96 ± 17.06	24.58 ± 18.91^∗∗^	25.66 ± 18.49^∗∗^	36.71 ± 24.23	26.55 ± 14.50	34.81 ± 20.47	29.06 ± 16.78	24.25 ± 14.85^∗^	25.64 ± 16.01^∗^	22.03 ± 15.64^∗∗^	25.76 ± 15.85^∗^
Kidney	25.05 ± 13.27	30.23 ± 15.88	22.52 ± 18.19	25.04 ± 18.77	28.65 ± 20.06	24.19 ± 15.74	28.51 ± 16.36	28.58 ± 15.32	19.40 ± 13.58^∗^	21.24 ± 13.87^∗^	20.46 ± 13.40^∗∗^	25.03 ± 16.22
Bladder	31.93 ± 12.37	32.93 ± 19.79	23.36 ± 16.94^∗∗^	24.32 ± 17.66^∗∗^	30.34 ± 20.63	25.76 ± 15.36^∗^	28.08 ± 13.87	25.09 ± 12.50	22.43 ± 13.50	19.84 ± 12.48^∗^	20.51 ± 12.52^∗^	20.91 ± 12.05^∗^
Gallbladder	25.49 ± 12.59	27.66 ± 15.55	21.10 ± 17.01	22.75 ± 17.33	28.31 ± 19.03	23.90 ± 13.04	28.51 ± 17.49	25.62 ± 15.65	18.82 ± 12.11^∗^	22.04 ± 14.84	19.74 ± 14.02^∗^	21.78 ± 11.85
Stomach	31.27 ± 13.08	30.94 ± 16.74	21.10 ± 17.01	25.76 ± 18.59	33.02 ± 20.83	26.85 ± 16.13	29.15 ± 18.75	25.70 ± 14.03	21.23 ± 12.39^∗^	23.63 ± 15.71^∗^	21.90 ± 13.76^∗^	21.72 ± 14.08^∗^

^∗^ *P* < 0.05 by paired-samples *t*-test.

^∗∗^ *P* < 0.01 by paired-samples *t*-test.

Data are expressed as means ± standard deviation.

**Table 3:** Comparison of the two groups in VAS in each treatment.

	Active group	Placebo group	*P* value
First day	5.00 ± 1.96	5.46 ± 1.96	0.214
Second day	4.60 ± 2.11	5.09 ± 2.12	0.182
Third day	4.03 ± 2.03	4.49 ± 1.96	0.126
Forth day	3.60 ± 1.58	3.85 ± 1.97	0.088
Fifth day	3.11 ± 1.54	3.20 ± 1.84	0.145

^∗∗^ *P* < 0.01 by paired-samples *t*-test.

**Table 4:** VAS comparison of active group.

	Active group		*P* value
	First day	Fifth day	
Before	6.25 ± 1.75	4.25 ± 1.72	0.000^∗∗^
After	5.00 ± 1.96	3.11 ± 1.54	0.000^∗∗^

^∗∗^ *P* < 0.01 by paired-samples *t*-test.

**Table 5:** VAS comparison of placebo group.

	Placebo group		*P* value
	First day	Fifth day	
Before	6.54 ± 2.05	3.72 ± 1.67	0.000^∗∗^
After	5.46 ± 1.96	3.20 ± 1.84	0.000^∗∗^

^∗∗^ *P* < 0.01 by paired-samples *t*-test.
